# Small airway function in predicting asthma control in preschool children

**DOI:** 10.1002/pdi3.46

**Published:** 2024-03-08

**Authors:** Liangqin Yi, Yan Zhao, Ziyao Guo, Qinyuan Li, Chunlan Qiu, Jingyi Yang, Sha Liu, Fangjun Liu, Ximing Xu, Zhengxiu Luo

**Affiliations:** ^1^ Department of Respiratory Medicine Children’s Hospital of Chongqing Medical University Chongqing China; ^2^ National Clinical Research Center for Child Health and Disorders Chongqing China; ^3^ Ministry of Education Key Laboratory of Child Development and Disorders Chongqing China; ^4^ Chongqing Key Laboratory of Pediatrics Chongqing China; ^5^ Department of Respiratory Medicine Pulmonary Function Test Room Children’s Hospital of Chongqing Medical University Chongqing China; ^6^ Big Data Center for Children's Medical Care Children's Hospital of Chongqing Medical University Chongqing China

**Keywords:** asthma control, FEF_50_, preschool children, small airway function

## Abstract

Asthma control in children is often challenging. This retrospective cohort study aimed to investigate the potential contribution of small airway function in predicting asthma control within a 2‐ to 3‐month period following the initial diagnosis in preschool children with asthma. A total of 219 preschool children diagnosed with asthma were enrolled, and their follow‐up was conducted by pediatric pulmonary physicians. Clinical history and lung function results were collected for analysis. To identify risk factors associated with poor asthma control, a multivariable regression model was employed. Sixty‐nine of the patients (31.5%) exhibited poor asthma control. Poor adherence to therapy (14.5% vs. 6.0%, *p* = 0.038) and the presence of severe airway hyperresponsiveness (AHR) (20.6% vs. 1.6%, *p < *0.001) were more prevalent in the group with poor control. Additionally, baseline forced expiratory volume in 1 s in predicting (94.5% vs. 101.4%, *p* = 0.001), forced expiratory flows (FEF)_50_% (66.1% vs. 86.0%, *p < *0.001), FEF_75_% (60.9% vs. 75.3%, *p* = 0.001), and FEF_25–75_% (70.9% vs. 86.0%, *p < *0.001) were significantly lower in the poorly‐controlled group than those of well‐controlled group. There was no significant difference in forced vital capacity in predicting (FVC%) between the two groups (92.4% vs. 96.7%, *p* = 0.093). Multivariable regression model unveiled initial severe AHR (OR 8.595, 95%CI 1.241–59.537, *p* = 0.021) and decreased FEF_50_% (OR 0.971, 95%CI 0.949–0.994, *p* = 0.012) were significantly associated with short‐term poor asthma control. Preschool children with asthma who exhibites initial severe AHR and/or decreased FEF_50_% faces an elevated risk of encountering poor asthma control during the subsequent 2–3 months.

## Graphical abstract

This study retrospectively analyzed the clinical history and lung function results to determine risk factors of short‐term poor asthma control. The findings revealed that preschool children with asthma who had severe airway hyperresponsiveness and/or decreased forced expiratory flow at 50% of forced vital capacity predicting (FEF_50_%) were at risk of poor asthma control in the subsequent 2–3 months.

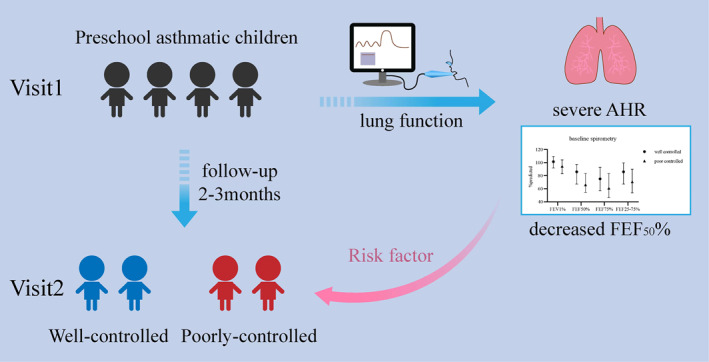

## 1 INTRODUCTION

Asthma represents a prevalent chronic condition affecting individuals of all age groups worldwide, with preschool children exhibiting the highest prevalence rate.^1^ While randomized controlled trials have demonstrated the achievability of asthma control,^2^ real‐world clinical practice frequently falls short in achieving optimal control levels. The prevalence rate of inadequately controlled asthma in children varies significantly ranging from 20% to 60%.^3^, ^4^

The evaluation of asthma control in children heavily relies on subjective reports provided by patients and their parents. However, differences in caregivers' comprehension of the disease can lead to over‐ or underestimation of symptoms, resulting in limited correlations between subjective reports and objective measurements.^5^ Hence, there is a critical necessity to identify dependable markers for assessing asthma control in children.

Forced expiratory volume in 1 s (FEV_1_) serves as the gold standard for clinically evaluating airway obstruction and treatment response.^6^ However, FEV_1_ primarily reflects large airway obstruction and may not be strongly associated with asthma control, particularly in pediatric patients.^7^, ^8^ Growing evidence underscores the pivotal role of small airways in asthma control.^9^, ^10^ Studies have indicated that both current symptoms and small airway dysfunction are predictive of future asthma exacerbations.^10^, ^11^ Spirometry measurements of forced expiratory flows (FEFs) taken at the mid‐portion of the flow‐volume loops, specifically forced expiratory flow at 50% of forced vital capacity (FVC) (FEF_50_), forced expiratory flow at 75% of FVC (FEF_75_), and forced expiratory flow between 25% and 75% of FVC (FEF_25__–__75_) provide effective means of assessing small airway function. These parameters are considered less dependent on patient effort compared to FEV1.^12^

Despite the significance of small airway function in forecasting asthma control, there has been a paucity of studies specifically examining its role in pediatric populations. The potential incorporation of small airway function into a comprehensive assessment of pediatric asthma control remains uncertain.^6^ Consequently, this study aims to explore the relevance of spirometry‐based small airway function measurements in predicting asthma control among preschool children with asthma.

## 2 METHODS

### 2.1 Patients

This retrospective cohort study obtained approval from the Institutional Review Board of the Children's Hospital of Chongqing Medical University (File No. [2022] 186). Due to the retrospective nature of the study, the necessity for informed consent was waived.

The study was conducted at the Children's Hospital of Chongqing Medical University, a 2000‐bed tertiary teaching hospital located in Chongqing, China. The research focused on preschool children who received an initial diagnosis of asthma and underwent two consecutive visits with pediatric pulmonologists between January 1, 2019, and December 31, 2020. Patients were included based on the following criteria: (i) age between 3 and 5 years old; (ii) diagnosis of asthma confirmed by at least one pediatric pulmonologist at the Children's Hospital of Chongqing Medical University, following the GINA guidelines (2018)^6^; (iii) completion of a standard lung function test with technically acceptable flow‐volume curves^13^; (iv) free from respiratory infections for a minimum of 4 weeks preceding the lung function test. Patients were excluded from the study if they met any of the following criteria: (i) presence of acute and/or chronic diseases that could potentially impact the results of the lung function test (including conditions like bronchopulmonary dysplasia, bronchiectasis, pulmonary tuberculosis, interstitial lung disease, congenital heart disease, severe psychiatric disorders, etc.); (ii) insufficient quality of spirometry data; (iii) lack of medical records for the 2–3 months of the follow‐up period.

### 2.2 Definitions

According to the GINA guidelines (2018),^6^ well‐controlled asthma is defined as the absence of the following conditions: (i) experiencing daytime asthma symptoms for more than a few minutes, more than once a week; (ii) encountering any limitations in activities, such as reduced running or playing compared to other children, or experiencing easy fatigue during physical activities due to asthma; (iii) requiring reliever medication more than once a week; (iv) experiencing nighttime awakenings or coughing due to asthma. Partly controlled asthma is defined as meeting 1 to 2 of the aforementioned conditions, and uncontrolled asthma is defined as meeting 3 to 4 of the mentioned conditions. For this study, poorly‐controlled asthma encompasses both partly controlled and uncontrolled asthma.

Children were classified as atopic types if they demonstrated at least one positive response to common aeroallergens (such as house dust mites, cotton, fur, etc.) or food allergens (such as peanut, milk, egg, etc.) during a skin prick test.^3^ Eosinophilia was defined as a peripheral blood eosinophil count equal to or greater than 0.5 × 10^9^/L with eosinophils constituting at least 5% of leukocytes.^14^

Poor adherence to therapy was defined as meeting any of the following criteria^15^: (i) not utilizing a medication controller device, (ii) employing an incorrect inhalation method, and (iii) consuming less than 80% of the prescribed doses.

### 2.3 Lung function

Spirometry (Masterscreen Pead, Germany JAEGER) was performed following the guidelines set forth by the American Thoracic Society (ATS) and/or the European Respiratory Society (ERS).^13^ Trained technicians administered the lung function tests within the dedicated laboratory setting. Each test underwent a minimum of three repetitions to ensure the reproducibility of both FVC and FEV_1_. The most successful FVC maneuver out of the three attempts was selected. A qualified investigator meticulously reviewed the volume‐time and flow‐volume tracings, excluding any measurements of subpar quality.

Airway hyperresponsiveness (AHR) was categorized into four degrees based on methacholine concentration (cMch) according to a previously published study^16^: Borderline AHR: 8 g/L* < *cMch ≤16 g/L, Mild AHR: 2 g/L* < *cMch ≤ 8 g/L, Moderate AHR: 0.5 g/L* < *cMch ≤ 2 g/L, Severe AHR: cMch ≤ 0.5 g/L.

### 2.4 Data collection

The data were obtained through a review of electronic medical databases of the Department of Respiratory Medicine. Two trained researchers independently extracted information from the medical records using a standardized data collection form. In cases where discrepancies arose, a third researcher was consulted for resolution. The following demographic characteristics were gathered: gender, age, weight, height, and body mass index (BMI), along with maternal and paternal histories of asthma, comorbidities (such as allergic rhinitis and eczema), peripheral eosinophil count and proportion, results of the skin prick test, and measurements of FVC, FEV1, FEFs, and cMch at baseline (Visit 1) and follow‐up (Visit 2). All spirometry parameter were expressed as percentages of predicted values (%pred).

### 2.5 Statistical analysis

The normality of continous variables was evaluated using the Shapiro‐Wilk test. Continuous variables were expressed as median and interquartile range, while categorical variables were presented as frequencies and percentages (%). Disparities in continuous variables between groups were assessed using the non‐parametric Mann‐Whitney *U* test. The paired Wilcoxon signed‐rank test was employed to analyze differences in continuous variables between the two consecutive visits within each group. Categorical variables were compared using the Chi‐square test supplemented by Fisher's exact test when necessary, and Bonferroni correction was applied to account for multiple comparisons. Interdependencies among variables (FVC%, FEV_1_%, and FEF_S_%) were assessed through collinearity diagnostics and Spearman's correlation analysis. The correlation coefficient |*r*| < 0.400 indicated a weak correlation, 0.400–0.700 suggested a moderate correlation, and >0.70 indicated a strong correlation.^17^ The logistic regression model was applied for multivariable analyses to determine independent factors associated with poor asthma control. Variables demonstrating significance with a *p*‐value <0.050 in univariate analysis were subsequently included in the multivariate logistic regression analysis using the entering selection method. Receiver‐operating characteristic (ROC) curves were constructed to evaluate the predictive performance of the models for asthma control. Independent variables, including age, BMI, FVC%, FEV1%, and FEF_S_%, were considered as continuous variables. Categorical variables, such as a history of allergic rhinitis, eczema, atopy and poor adherence to therapy, were coded as 1 (present) or 0 (not present). AHR was categorized into four degrees and coded as 0 (borderline), 1 (mild), 2 (moderate), and 3 (severe) for the analysis. All data analyses were conducted by IBM SPSS software for Windows (version 26.0 SPSS Inc.), and *p*‐values less than 0.050 (two‐tailed) were considered statistically significant.

## 3 RESULTS

### 3.1 Characteristics of children with poor asthma control

**FIGURE 1:**
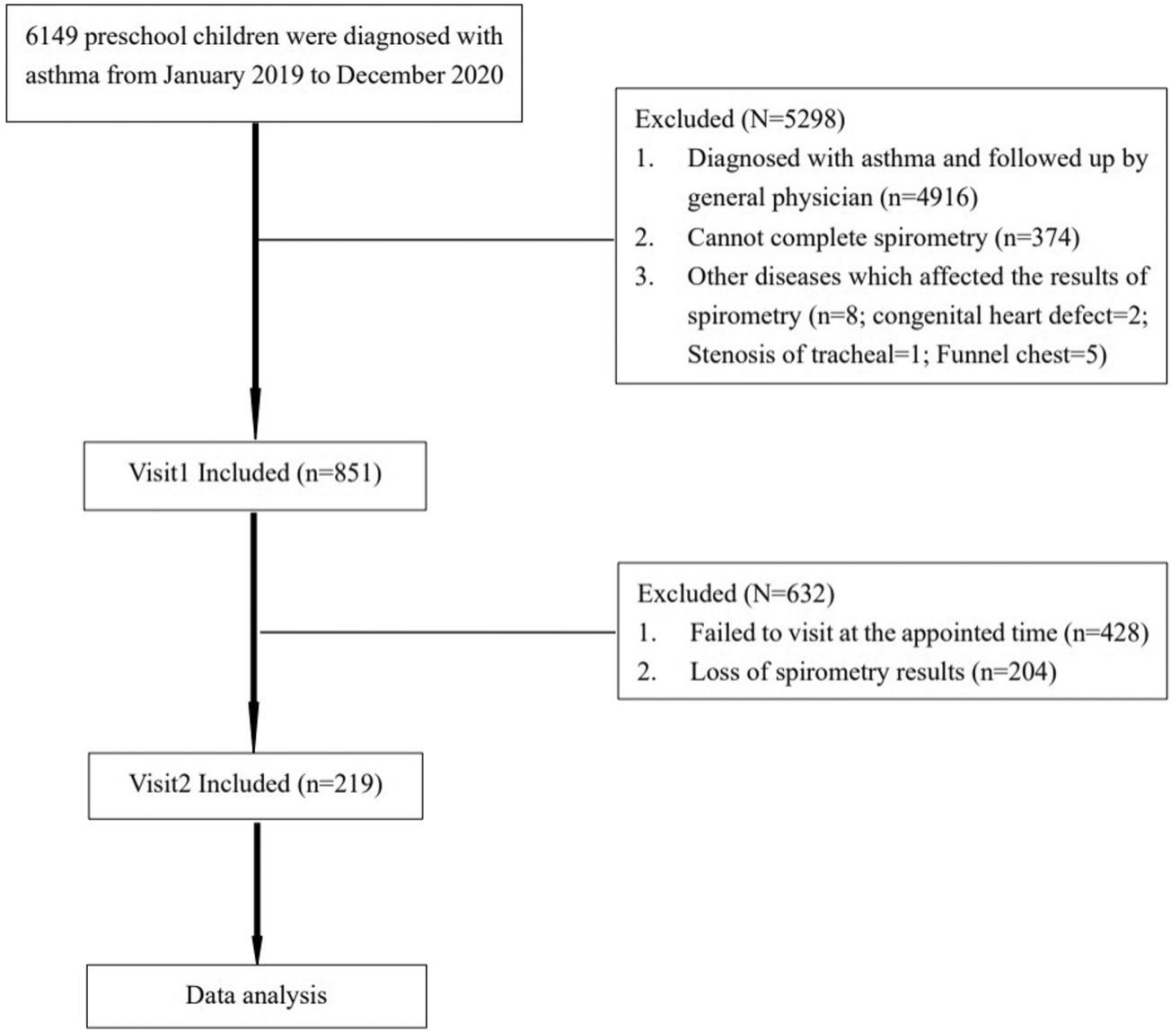
Flow diagram of study progression.

In this retrospective cohort study, a total of 851 preschool children who had confirmed asthma at the initial visit (Visit 1) met the inclusion criteria. Among them, 428 patients did not attend the follow‐up visit within the subsequent 2–3 months (Visit 2), and spirometry data of 204 patients were missing. Consequently, 219 preschool children with asthma were included in the study (see Figure 1). The median age of the participants was 4.4 years. Out of the 219 patients, 150 (68.5%) achieved good asthma control, while 69 (31.5%) experienced poor asthma control. Children in the poorly‐controlled group were slightly younger than those in the well‐controlled group (4.3 years vs. 4.5 years, *p* = 0.049). The prevalence of poor adherence (14.5% vs. 6.0%, *p* = 0.038) and severe AHR (20.6% vs. 1.6%, *p < *0.001) were remarkably higher in the poorly‐controlled group than the well‐controlled group. However, there were no statistically significant differences in terms of gender (*p* = 0.558), BMI (*p* = 0.766), family history of asthma (*p* = 0.157), eczema (*p* = 0.110), allergic rhinitis (*p* = 0.696), eosinophilia (*p* = 0.719), atopic status (*p* = 0.397), and medication management (*p* = 0.626) between the two groups (Table 1).

**TABLE 1:** Baseline characteristics in groups.

	Total (*n* = 219)	Well‐controlled (*n* = 150)	Poorly‐controlled (*n* = 69)	*p* value (well‐controlled vs. poorly‐controlled)
Age of onset (y)	4.4 (3.9–5.0)	4.5 (4.0–5.3)	4.3 (3.9–4.7)	0.049
Male sex	127 (58.0%)	85 (56.7%)	42 (60.9%)	0.558
BMI	15.9 (15.0–16.8)	15.8 (15.1–16.8)	15.9 (14.8–16.8)	0.766
Family asthmatic history	18 (8.2%)	15 (10.0%)	3 (4.3%)	0.157
Eosinophilia	53 (24.2%)	36 (24.0%)	17 (24.6%)	0.719
Allergic rhinitis	110 (50.2%)	74 (49.3%)	36 (52.2%)	0.696
Eczema	60 (27.4%)	46 (30.7%)	14 (20.3%)	0.110
Atopy	124 (56.6%)	85 (56.7%)	39 (56.5%)	0.397
AHR	190 (86.8%)	127 (84.7%)	63 (91.3%)	<0.001
Borderline	16 (7.3%)	11 (7.3%)^†^	5 (7.2%)^†^	
Mild	64 (29.2%)	44 (29.3%)^†^	20 (29.0%)^†^	
Moderate	95 (43.4%)	70 (46.7%)^†^	25 (36.2%)^‡^	
Severe	15 (6.8%)	2 (1.3%)^†^	13 (18.8%)^‡^	
LTRAs/ICS	9/210	5/145	4/65	0.626
Poor adherence to therapy	19 (8.7%)	9 (6.0%)	10 (14.5%)	0.038

*Note*: *p*‐value calculated by the chi‐square test (for categorical variables) or Mann‐Whitney *U* test (for continuous variables). Symbols ^†^, ^‡^ in the same row, different symbols indicate a significant difference between the groups (*p* < 0.050); same symbols indicate no difference between groups (*p* > 0.050).

Abbreviations: AHR: airway hyperresponsiveness; BMI, body mass index; ICS: inhaled corticosteroids; LTRAs: leukotriene receptor antagonists.

### 3.2 Decreased lung function was associated with poor asthma control in preschool children with asthma

At the baseline, the poorly‐controlled group exhibited significantly lower values for FEV_1_% (94.5 vs. 101.4, *p* = 0.001), FEF_50_% (66.1 vs. 86.0, *p < *0.001), FEF_75_% (60.9 vs. 75.3, *p* = 0.001), and FEF_25–75_% (70.9 vs. 86.0, *p < *0.001) compared to the well‐controlled group. However, there was no statistically significant difference in FVC% (92.4 vs. 96.7, *p* = 0.093) between the two groups. Throughout the study, spanning from visit 1 to visit 2, spirometry parameters, including FEV_1_%, FEF_50_%, FEF_75_%, and FEF_25–75_% exhibited statistically significant improvements in both groups. Nevertheless, the extent of improvement in these spirometry parameters did not differ significantly between the two groups. For detailed data, please refer to Table 2.

**TABLE 2:** Comparison of spirometry parameters between well‐controlled and poorly‐controlled groups.

	Visit 1			Visit 2		
	Well‐controlled (150)	Poorly‐controlled (69)	*p* value^a^	Well‐controlled (150)	Poorly‐controlled (69)	*p* value^a^
FVC%	96.7 (87.0–105.3)	92.4 (83.7–103.5)	0.093	98.2 (90.8–107.1)	95.3 (86.6–99.7)	0.009
FEV_1_%	101.4 (91.9–109.5)	94.5 (83.0–104.3)	0.001	104.3 (96.8–112.5)^†^	98.0 (91.8–106.3)^†^	0.001
FEF_50_%	86.0 (67.3–97.4)	66.1 (54.4–83.4)	<0.001	94.3 (74.7–108.9)^‡^	79.6 (65.6–97.6)^‡^	0.001
FEF_75_%	75.3 (57.3–92.7)	60.9 (46.4–83.4)	0.001	85.0 (68.5–103.9)^‡^	69.9 (55.8–95.9)^†^	<0.001
FEF_25–75_%	86.0 (67.2–99.7)	70.9 (53.9–89.8)	<0.001	95.7 (77.0–109.1)^‡^	78.3 (66.3–95.1)^†^	<0.001

*Note*: The paired Wilcoxon signed‐rank test was used to test differences between visits within groups: ^†^ *p* ≤ 0.050, ^‡^ *p* ≤ 0.001.

Abbreviations: FVC%, forced vital capacity in predicting; FEV_1_%, forced expiratory volume in 1 s in predicting; FEF_50_%, forced expiratory flow at 50% of FVC predicting; FEF_75_%, forced expiratory flow at 75% of FVC predicting; FEF_25–75_%, forced expiratory flow between 25% and 75% of FVC predicting.

^a^ The Mann‐Whitney *U* test was applied to test the difference within each group on two visits.

### 3.3 Risk factors in predicting poor asthma control in preschool children with asthma

The univariable analysis revealed that factors, such as the age of onset, adherence, baseline FEV_1_%, FEF_50_%, FEF_75_%, FEF_25–75_%, as well as AHR, were associated with short‐term poor asthma control in preschool children with asthma (*p < *0.050 for each factor, detailed information can be found in Supplement Table 1). Collinearity diagnostics identified interdependencies among these variables, notably a strong correlation between baseline FEF_50_% and FEF_75_% (correlation coefficient: 0.872, *p* < 0.010) and between baseline FEF_50_% and FEF_25–75_% (correlation coefficient: 0.963, *p* < 0.010, refering to Supplement Table 2 for detailed information). Given these strong correlations among FEFs, it was advisable to include only one of them in the logistic regression model at a time. Therefore, we conducted three separate multivariate regression models to determine the independent predictors of poor asthma control at visit 2 (Model 1 included age, adherence, AHR, baseline FEV_1_, and baseline FEF_50_%; Model 2 included age, adherence, AHR, baseline FEV_1_, and baseline FEF_75_%; Model 3 included age, adherence, AHR, baseline FEV_1_, and baseline FEF_25–__75_%. Please refer to Table 3 for details).

**TABLE 3:** Multivariable logistic regression analysis for subsequent 2–3 months poor asthma control.

Variables	OR	95%CI	*p* value
Model 1			
Age of onset (y)	0.574	0.348–1.946	0.129
Adherence	2.224	0.734–6.736	0.157
AHR^a^			0.021
Borderline (reference)			
Mild	0.950	0.264–3.418	
Moderate	0.640	0.183–2.242	
Severe	8.595	1.241–59.537	
Baseline FEV_1_%	0.995	0.961–1.030	0.779
Baseline FEF_50_%	0.971	0.949–0.994	0.012
Model 2			
Age of onset (y)	0.534	0.321–1.889	0.116
Adherence	2.452	0.805–7.466	0.114
AHR^a^			0.021
Borderline (reference)			
Mild	0.978	0.275–3.475	
Moderate	0.652	0.189–2.251	
Severe	8.393	1.239–56.844	
Baseline FEV_1_%	0.983	0.952–1.015	0.294
Baseline FEF_75_%	0.983	0.966–1.001	0.061
Model 3			
Age of onset (y)	0.559	0.339–1.921	0.123
Adherence	2.320	0.765–7.032	0.137
AHR^a^			0.022
Borderline (reference)			
Mild	0.955	0.269–3.395	
Moderate	0.655	0.189–2.263	
Severe	8.551	1.256–58.220	
Baseline FEV_1_%	0.988	0.954–1.023	0.505
Baseline FEF_25–75_%	0.979	0.958–1.000	0.055

*Note*: Model 1: including age, adherence, AHR, baseline FEV_1_%, and FEF_50_%; Model 2: including age, adherence, AHR, baseline FEV_1_%, and FEF_75_%; Model 3: including age, adherence, AHR, baseline FEV_1_%, and FEF_25–75_%.

Abbreviations: AHR, airway hyperresponsiveness; FEV_1_%, forced expiratory volume in 1 s in predicting; FEF_50_%, forced expiratory flow at 50% of forced vital capacity (FVC) predicting; FEF_75_%, forced expiratory flow at 75% of FVC predicting; FEF_25–75_%, forced expiratory flow between 25% and 75% of FVC predicting; FVC, forced expiratory vital capacity; *p* values were calculated by multivariable regression models.

^a^ AHR was transformed into three dummy variables, and the borderline AHR was administered as a reference.

**FIGURE 2:**
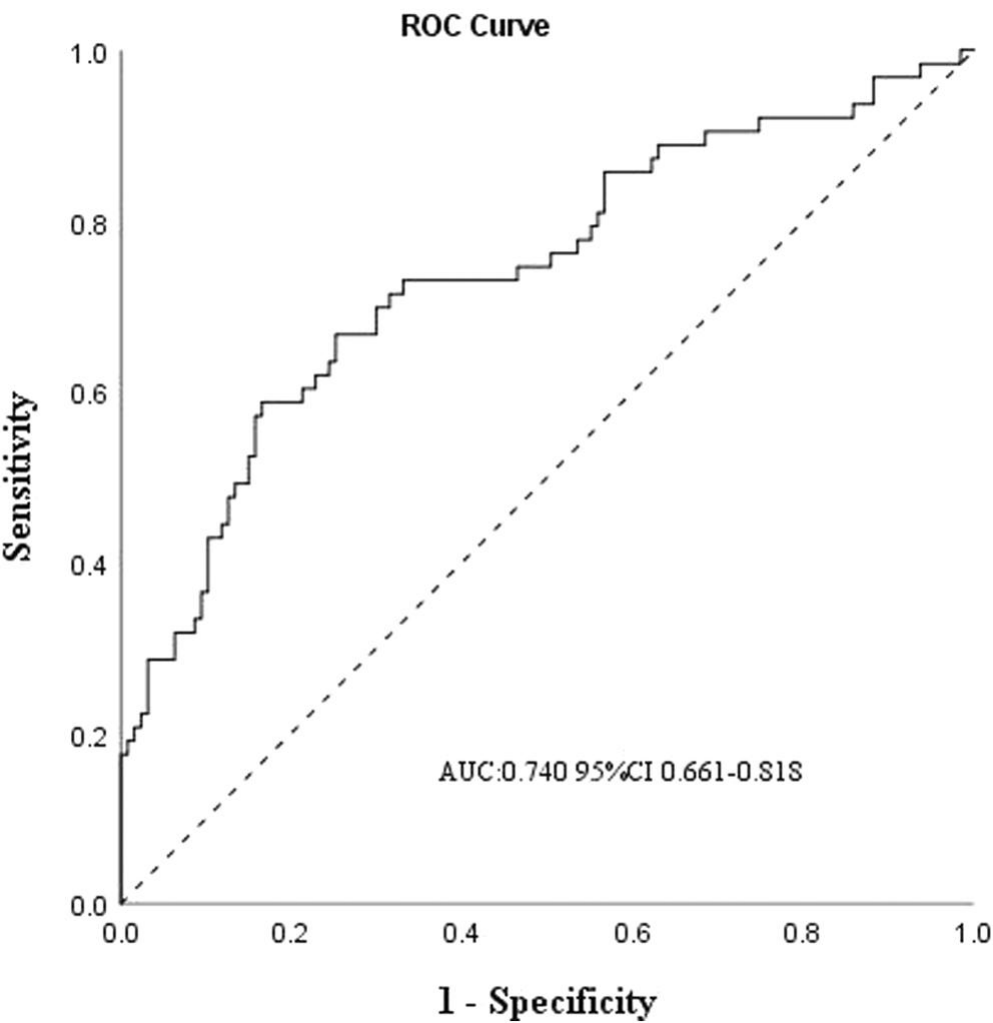
ROC of the model in predicting subsequent 2‐ to 3‐months of poorly‐controlled asthma. A model including baseline AHR and FEF_50_%. AHR, airway hyperresponsiveness; AUC, area under the curve; FEF_50_%, forced expiratory flow at 50% of forced vital capacity predicting; ROC, receiver‐operating characteristic.

The predictive model for poor asthma control in preschool children with asthma over the next 2–3 months revealed that baseline severe AHR (OR 8.595, 95%CI 1.241–59.537, *p* = 0.029) and decreased FEF_50_% (OR 0.971, 95%CI 0.949–0.994, *p* = 0.012) were significantly associated with short‐term poor asthma control (Table 3). Preschool children with asthma who had baseline severe AHR were approximately 9 times more likely to experience poor asthma control within the next 2–3 months compared to those with baseline borderline AHR. Additionally, for each unit decrease in baseline FEF_50_%, the odds of future poor asthma control increased by 3% in preschool children with asthma (Table 3). The ROC curve demonstrated that the multivariable model, which included baseline AHR and FEF_50_%, exhibited good predictive ability for poor asthma control in preschool children with asthma (area under the curve: 0.740, 95%CI 0.661–0.818; Figure 2).

## 4 DISCUSSION

Our study aimed to investigate the correlation between current clinical status, spirometry parameters, and asthma control in preschool children with asthma over a 2‐ to 3‐month period. The results revealed that severe AHR and a lower FEF_50_% value at the initial visit were significantly linked to poor asthma control during the subsequent 2–3 months.

Recent research has increasingly emphasized the pivotal role of small airways in the pathogenesis of asthma. Studies consistently indicate that inflammatory cell infiltration and airflow limitation predominantly manifest in these smaller airways.^18^, ^19^ The persistence of inflammation within these small airways has been identified as a primary contributor to poor asthma control.^20^ The results of our study align seamlessly with these findings as we have demonstrated that a spirometric parameter reflecting small airway function serves as a significant predictor of future asthma control in preschool children. Notably, our findings corroborate a study conducted by Shi et al.,^10^ providing additional compelling evidence for the relevance of small airway function in predicting asthma outcomes.

Remarkably, our multivariate analysis uncovered that an initial decrease in FEF_50_% played a significant role in poor asthma control, while the impact of FEV_1_% on asthma control within the model was not as pronounced. Previous studies have reported relatively weak correlations between FEV_1_ and symptoms, respiratory status, and dyspnea in patients with asthma.^9^ In contrast, small airway function has been associated with nocturnal asthma, heightened asthma symptoms, and exercise‐induced asthma.^21^ Moreover, airway dysfunction in children with asthma is not uniformly distributed throughout the airways^22^ with inflammation often being more evident in the small airways than the larger ones.^18^ The decline in FEFs may be more common in the early stage of childhood before a noticeable decrease in FEV_1_. This suggested that preschool patients with decreased small airway function may be at a heightened risk of asthma exacerbation.^8^ Furthermore, Turner's study has indicated that FEV_1_ in the majority of preschool children with asthma falls within the normal range, and FEV_1_ may not be sufficiently sensitive for detecting abnormalities in asymptomatic individuals.^7^ In our study, despite a significant improvement in spirometry parameters, including FEV_1_% >80%, in the poorly‐controlled group's following treatment, varying degrees of impairment in small airway function persisted. This highlights the limited capacity of FEV_1_ to effectively gauge asthma control and airflow limitation in preschool children. Conversely, small airway function parameters demonstrate greater reliability in reflecting airway dysfunction and predicting poor asthma control. In contrast, FEV_1_ and FEFs have shown a significant association with the Asthma Control Questionnaire in adults.^9^ This difference may be attributed to increased asthma duration, the persistence of airway inflammation, and airway remodeling occurring in both large and small airways with age, ultimately leading to airflow limitation. Thus, this highlights the importance of considering both large and small airways when assessing asthma control.

In 1975, Dosman's study demonstrated that a decrease in FEF_50_ was a sensitive method for detecting small airway obstruction.^23^ The validation study of ATS/ERS spirometry guidelines also showed high sensitivity and specificity for FEF_50_ in identifying airway obstruction in 5‐year‐old children with asthma.^7^ Our results further reinforce the role of decreased baseline FEF_50_% as a risk factor for short‐term poor asthma control. However, the debate surrounding FEF_50_ versus FEF_25–75_ in assessing airway function in asthma, particularly in children, remains unresolved.^24^, ^25^ We acknowledge the significant contribution of small airway function to asthma control. However, in our study, we observed that despite these improvements in spirometry parameters, some patients continued to experience poor asthma control. This finding underscored the complexity of asthma and suggested that while spirometry parameters like FEF_50_% were important components of asthma control assessment, they alone may not provide a comprehensive representation of asthma control. Asthma control is influenced by a multitude of factors, and our study highlighted the importance of considering a comprehensive set of clinical and physiological variables when assessing asthma outcomes. As spirometry is a convenient and practical method for evaluating airflow limitation, further studies exploring the impact of FEFs on airway function in preschool children with asthma would be both valuable and necessary, especially in economically disadvantaged areas where more advanced pulmonary function tests, such as impulse oscillometry (IOS), fractional exhaled nitric oxide (FENO), and multiple breath nitrogen washout (MBNW), may not be readily available.

Contrary to other studies,^26^, ^27^ our study did not identify a significant relationship between rhinitis, eczema, family asthmatic history, and short‐term asthma control in preschool children with asthma. These discrepancies may be attributed to variations in the study population, sample size, and follow‐up periods. Results on adherence were also found to be heterogeneous,^27^, ^28^ potentially due to the relatively brief follow‐up period or improvements in lung function resulting from treatment, which may overshadow the influence of adherence on asthma control. Nonetheless, emphasizing good adherence remains crucial for achieving optimal asthma control.

AHR is a fundamental component of asthma pathophysiology^6^ and is closely linked to heightened airway inflammation.^29^ Evidence suggests that asymptomatic AHR in the general population serves as an important risk factor for the subsequent development of wheezing or asthma.^30^ Furthermore, severe AHR in infancy has been identified as a predictor of asthma development in adulthood.^31^ Our study further underscored the significance of severe AHR as a risk factor for future poor asthma control, emphasizing its potential as a predictor of asthma outcomes. Nevertheless, additional research is needed to confirm the relevance of AHR in the development of asthma.

In summary, our study revealed that initial severe AHR and decreased FEF_50_ are associated with short‐term poor asthma control in preschool children. By incorporating these indicators into routine assessments, healthcare providers can identify high‐risk individuals who may require personalized treatments. This approach holds the potential to optimize asthma control and reduce the likelihood of exacerbations.^32^, ^33^ The regular monitoring of AHR and FEF50 may aid in the early identification of asthma in preschool children who are at risk of poor asthma control, enabling the timely implementation of tailored treatments. Additionally, advancements in technology may lead to the development of portable monitoring devices, such as home electronic peak flowmeters, which could broaden the application of these indicators in various settings. This, in turn, would provide clearer guidance to parents and caregivers regarding medication use, trigger avoidance, and the prompt recognition of worsening symptoms. Furthermore, ongoing research endeavors are essential for enhancing the utilization of non‐invasive tests, ultimately improving the care and outcomes for preschool children with asthma.

### 4.1 Strength and limitation

Our study has provided valuable insights into the relationship between small airway function and asthma control in preschool children, specifically highlighting the association between decreased small airway function and poor asthma control in the 2–3 months following the initial diagnosis. However, we must acknowledge several limitations. First, there was a potential risk of collinearity between FEV_1_% and the FEFs%, which may weaken the statistical analysis. Although we addressed multicollinearity through residual analysis and conducted sensitivity analysis (see Supplement Tables S3–S7), caution is warranted when comparing the predictive value of FEV_1_ and the FEFs in assessing asthma control. Second, due to the overlap of the COVID‐19 pandemic with our study period, a significant number of patients were lost to follow‐up, and the follow‐up duration was relatively short, which could pose challenges in interpreting certain results. Additionally, the retrospective design and relatively small sample size may limit the generalizability of our findings. Therefore, further prospective studies with larger cohorts are necessary to validate these results. Furthermore, the 2‐ to 3‐month follow‐up period may not capture longer‐term asthma control outcomes, necessitating future research to investigate the predictive value of these factors over an extended follow‐up duration. Moreover, our study exclusively focused on spirometry parameters when assessing the correlation between small airway function and asthma control without considering other pulmonary function tests, such as IOS, FENO, and MBNW. Given the varying sensitivity of different methods, combining multiple techniques to assess small airway function may offer additional information for predicting asthma control. Lastly, in our study cohort, some preschool children with poor asthma control continued to exhibit small airway dysfunction despite receiving standard therapy. Further research is required to determine the prognostic role of persistent small airway dysfunction in asthma.

## 5 CONCLUSION

The study aimed to identify factors associated with asthma control in preschool children with asthma, providing valuable predictive information for poor asthma control. The results indicate that preschool children with asthma who have initial severe AHR and/or decreased FEF_50_ are at an elevated risk of experiencing poor asthma control over the subsequent 2–3 months. These findings underscore the importance of assessing small airway function in predicting asthma control and suggest that improving small airway function may be advantageous for managing asthma in this age group. Further research is essential to confirm these results and investigate additional factors that could impact asthma control in preschool children.

## AUTHOR CONTRIBUTIONS

**Zhengxiu Luo**: Conceptualization; supervision; writing—review and editing. **Liangqin Yi**: Conceptualization; data curation; methodology; formal analysis; writing—original draft preparation. **Yan Zhao**: Methodology; writing—review & editing. **Ziyao Guo**: Writing—review and editing. **Qinyuan Li**: Formal analysis; writing—review and editing. **Chunlan Qiu**: Investigation. **Jingyi Yang**: Investigation. **Sha Liu**: Data curation; investigation. **Fangjun Liu**: Data curation; investigation. **Ximing Xu**: Formal analysis; writing—review and editing.

## CONFLICT OF INTEREST STATEMENT

All authors declare they have no competing interests.

## ETHICS STATEMENT

This study was approved by the Institutional Review Board of the Children's Hospital of Chongqing Medical University (File No. [2022] 186). The requirement of obtaining informed consent was waived due to the retrospective design of the study.

## CONSENT TO PARTICIPATE

Not applicable.

## CONSENT FOR PUBLICATION

Not applicable.

## Supporting information

Table S1–S7

Table S8

## Data Availability

Data are available on request to the corresponding author.

## References

[1] Report A . Premium Times. November 1, 2022. Accessed May 5, 2021. https://www.premiumtimesng.com/news/top‐news/459514‐world‐asthma‐day‐339‐million‐people‐affected‐globally‐expert.html#.Y2pxApbQSC4.link

[2] Bateman ED , Boushey HF , Bousquet J , et al. Can guideline‐defined asthma control be achieved? The Gaining Optimal Asthma Control study. Am J Respir Crit Care Med. 2004;170(8), 836‐844. 15256389 10.1164/rccm.200401-033OC

[3] Papwijitsil R , Pacharn P , Areegarnlert N , et al. Risk factors associated with poor controlled pediatric asthma in a university hospital. Asian Pac J Allergy Immunol. 2013;31(3):253. 24053709

[4] Bao Y , Chen Z , Liu E , Xiang L , Zhao D , Hong J . Risk factors in preschool children for predicting asthma during the preschool age and the early school age: a systematic review and meta‐analysis. Curr Allergy Asthma Rep. 2017;17(12):85. 10.1007/s11882-017-0753-7 29151195

[5] Green RJ , Klein M , Becker P , et al. Disagreement among common measures of asthma control in children. Chest. 2013;143(1):117‐122. 22878380 10.1378/chest.12-1070

[6] Asthma GIf . Global Strategy for Asthma Management and Prevention. 2018. www.ginasthma.org

[7] Turner SW , Craig LC , Harbour PJ , et al. Spirometry in 5‐year‐olds—validation of current guidelines and the relation with asthma. Pediatr Pulmonol. 2007;42(12):1144‐1151. 17968994 10.1002/ppul.20709

[8] McFadden E Jr. , Linden DA . A reduction in maximum mid‐expiratory flow rate. A spirographic manifestation of small airway disease. Am J Med. 1972;52(6):725‐737. 5030170 10.1016/0002-9343(72)90078-2

[9] Takeda T , Oga T , Niimi A , et al. Relationship between small airway function and health status, dyspnea and disease control in asthma. Respiration. 2010;80(2):120‐126. 19776554 10.1159/000242113

[10] Shi Y , Aledia AS , Galant SP , George SC . Peripheral airway impairment measured by oscillometry predicts loss of asthma control in children. J Allergy Clin Immunol. 2013;131(3):718‐723. 23146376 10.1016/j.jaci.2012.09.022

[11] Robroeks CM , van Vliet D , Jöbsis Q , et al. Prediction of asthma exacerbations in children: results of a one‐year prospective study. Clin Exp Allergy. 2012;42(5):792‐798. 22515395 10.1111/j.1365-2222.2012.03992.x

[12] Postma DS , Brightling C , Baldi S , et al. Exploring the relevance and extent of small airways dysfunction in asthma (ATLANTIS): baseline data from a prospective cohort study. Lancet Respir Med. 2019;7(5):402‐416. 30876830 10.1016/S2213-2600(19)30049-9

[13] Miller MR , Hankinson J , Brusasco V , et al. Standardisation of spirometry. Eur Respir J. 2005;26(2):319‐338. 16055882 10.1183/09031936.05.00034805

[14] Knihtilä H , Kotaniemi‐Syrjänen A , Pelkonen AS , Mäkelä MJ , Malmberg LP . Small airway function in children with mild to moderate asthmatic symptoms. Ann Allergy Asthma Immunol. 2018;121(4):451‐457. 30059790 10.1016/j.anai.2018.07.026

[15] Mäkelä MJ , Backer V , Hedegaard M , Larsson K . Adherence to inhaled therapies, health outcomes and costs in patients with asthma and COPD. Respir Med. 2013;107(10):1481‐1490. 10.1016/j.rmed.2013.04.005 23643487 10.1016/j.rmed.2013.04.005

[16] Yuan H . Exploratory the Establishment of Improved Method in Bronchial Provocation Tests for Infants and the Clinical Application. Master. Chongqing Medical University; 2007. http://kns.cnki.net.forest.vpn358.com/KCMS/detail/detail.aspx?dbname=CMFD2008&filename=2007218528.nh

[17] Gudmund R , Iversen MG , Xizhi W . Statistics. China Higher Education Press/Springer Verlag; 2000.

[18] Braido F , Scichilone N , Lavorini F , et al. Manifesto on small airway involvement and management in asthma and chronic obstructive pulmonary disease: an Interasma (Global Asthma Association – GAA) and World Allergy Organization (WAO) document endorsed by Allergic Rhinitis and its Impact on Asthma (ARIA) and Global Allergy and Asthma European Network (GA(2)LEN). World Allergy Organ J. 2016;9:1‐6. 27800118 10.1186/s40413-016-0123-2 PMC5084415

[19] Bonser LR , Erle DA‐O . Airway mucus and asthma: the role of MUC5AC and MUC5B. J Clin Med. 2017;6(12):112. 29186064 10.3390/jcm6120112 PMC5742801

[20] Tirakitsoontorn P , Crookes M , Fregeau W , et al. Recognition of the peripheral airway impairment phenotype in children with well‐controlled asthma. Ann Allergy Asthma Immunol. 2018;121(6):692‐698. 30194972 10.1016/j.anai.2018.08.023

[21] Kraft M , Cairns C , Ellison MC , Pak J , Irvin C , Wenzel S . Improvements in distal lung function correlate with asthma symptoms after treatment with oral montelukast. Chest. 2006;130(6):1726‐1732. 17166989 10.1378/chest.130.6.1726

[22] Komarow HD , Skinner J , Young M , et al. A study of the use of impulse oscillometry in the evaluation of children with asthma: analysis of lung parameters, order effect, and utility compared with spirometry. Pediatr Pulmonol. 2012;47(1):18‐26. 22170806 10.1002/ppul.21507 PMC3423092

[23] Dosman J , Bode F , Urbanetti J , Martin R , Macklem PT . The use of a helium‐oxygen mixture during maximum expiratory flow to demonstrate obstruction in small airways in smokers. J Clin Invest. 1975;55(5):1090‐1099. 16695964 10.1172/JCI108010 PMC301856

[24] Murray AB , Ferguson AC . A comparison of spirometric measurements in allergen bronchial challenge testing. Clin Exp Allergy. 1981;11(1):87‐93. 10.1111/j.1365-2222.1981.tb01570.x 7214687

[25] Lutfi M , My S . Reliability of spirometric measurements in assessing asthma severity. Khartoum Med J. 2010;3:433‐439.

[26] Leiria‐Pinto P , Carreiro‐Martins P , Peralta I , et al. Factors associated with asthma control in 121 preschool children. J Investig Allergol Clin Immunol. 2021;31(6):471‐480. 10.18176/jiaci.0630 32694095

[27] Scott L , Morphew T , Bollinger ME , et al. Achieving and maintaining asthma control in inner‐city children. J Allergy Clin Immunol. 2011;128(1):56‐63. 21531451 10.1016/j.jaci.2011.03.020

[28] Gruchalla RS , Sampson HA , Matsui E , et al. Asthma morbidity among inner‐city adolescents receiving guidelines‐based therapy: role of predictors in the setting of high adherence. J Allergy Clin Immunol. 2009;124(2):213‐221. 19615730 10.1016/j.jaci.2009.05.036 PMC2757267

[29] Lötvall J , Inman M , O'Byrne P . Measurement of airway hyperresponsiveness: new considerations. Thorax. 1998;53(5):419‐424. 9708238 10.1136/thx.53.5.419 PMC1745221

[30] Brutsche MH , Downs SH , Schindler C , et al. Bronchial hyperresponsiveness and the development of asthma and COPD in asymptomatic individuals: SAPALDIA cohort study. Thorax. 2006;61(8):671‐677. 16670173 10.1136/thx.2005.052241 PMC2104688

[31] Palmer LJ , Rye PJ , Gibson NA , Burton PR , Landau LI , Lesouëf PN . Airway responsiveness in early infancy predicts asthma, lung function, and respiratory symptoms by school age. Am J Respir Crit Care Med. 2001;163(1):37‐42. 11208623 10.1164/ajrccm.163.1.2005013

[32] Martin RJ . Therapeutic significance of distal airway inflammation in asthma. J Allergy Clin Immunol. 2002;109(2 Suppl):S447‐S460. 11842317 10.1067/mai.2002.121409

[33] Abdo M , Watz H , Veith V , et al. Small airway dysfunction as predictor and marker for clinical response to biological therapy in severe eosinophilic asthma: a longitudinal observational study. Respir Res. 2020;21(1):278. 33087134 10.1186/s12931-020-01543-5 PMC7579879

