# Supplementary material for: Small airway function in predicting asthma control in preschool children
Source: Pediatr Discov. 2024 Mar 8;2(1):e46. doi: 10.1002/pdi3.46 (PMC12118259; doi:10.1002/pdi3.46)
Supplement: Supplementary file 1 — Table S1–S7 [file PDI3-2-e46-s002.docx]

| Supplement Table 1: Univariable logistic regression analysis for following 2 to 3 months of poor asthma control. | | | |
| --- | --- | --- | --- |
| Variables | OR | 95%CI | *P* value |
| Age of onset | 0.645 | 0.426-0.978 | 0.039 |
| Male sex | 1.190 | 0.665-2.128 | 0.558 |
| BMI | 1.003 | 0.833-1.208 | 0.971 |
| Family asthmatic history | 0.409 | 0.114-1.463 | 0.169 |
| Eosinophilia | 0.872 | 0.412-1.843 | 0.719 |
| Allergic rhinitis | 1.120 | 0.633-1.982 | 0.696 |
| Eczema | 0.575 | 0.291-1.138 | 0.112 |
| Atopy | 0.739 | 0.367-1.488 | 0.397 |
| AHR^*^ |  |  |  |
| borderline (reference) | 1.000 |  |  |
| mild | 1.000 | 0.307-3.261 | 1.000 |
| moderate | 0.786 | 0.248-2.485 | 0.681 |
| severe | 14.300 | 2.303-88.776 | 0.004 |
| Poor adherence to therapy | 2.655 | 1.026-6.869 | 0.044 |
| FVC% | 0.983 | 0.963-1.003 | 0.092 |
| FEV_1_% | 0.974 | 0.956-0.992 | 0.006 |
| FEF_50_% | 0.974 | 0.961-0.988 | ＜0.001 |
| FEF_75_% | 0.982 | 0.971-0.994 | 0.003 |
| FEF_25-75_% | 0.979 | 0.966-0.991 | 0.001 |
| Abbreviations: BMI, Body mass index; AHR, Airway hyperresponsiveness. FVC%, forced vital capacity in predicting; FEV_1_%, forced expiratory volume in 1 s in predicting; FEF_50_%, forced expiratory flow at 50% of FVC predicting; FEF_75_%, forced expiratory flow at 75% of FVC predicting; FEF_25-75_%, forced expiratory flow between 25% and 75% of FVC predicting.  Note: *AHR was transformed into three dummy variables, the borderline AHR was administered as a reference. *P*-value calculated by univariable logistic regression analysis. | | | |

| Supplement Table 2. Correlations of spirometry parameters. | | | | |
| --- | --- | --- | --- | --- |
|  | Baseline FVC%  r | Baseline FEV_1_%  r | Baseline FEF_50_%  r | Baseline FEF_75_%  r |
| Baseline FVC% |  |  |  |  |
| Baseline FEV_1_% | 0.841^**^ |  |  |  |
| Baseline FEF_50_% | 0.296^**^ | 0.677^**^ |  |  |
| Baseline FEF_75_% | 0.202^*^ | 0.629^**^ | 0.872^**^ |  |
| Baseline FEF_25-75_% | 0.276^**^ | 0.684^**^ | 0.963^**^ | 0.949^**^ |
| Abbreviations: FVC%, forced vital capacity in predicting; FEV_1_%, forced expiratory volume in 1 s in predicting; FEF_50_%, forced expiratory flow at 50% of FVC predicting; FEF_75_%, forced expiratory flow at 75% of FVC predicting; FEF_25-75_%, forced expiratory flow between 25% and 75% of FVC predicting.  Note: Spearman’s correlation was used for this analysis: **p*＜0.050, ***p*＜0.010. | | | | |

| Supplement Table 3. Multivariable logistic regression analysis for following 2 to 3 months of poor asthma control. | | | |
| --- | --- | --- | --- |
|  | OR | 95%CI | *P* |
| Severe AHR^*^ | 9.459 | 1.404-63.731 | 0.021 |
| Adherence | 2.025 | 0.682-6.012 | 0.204 |
| Unstandardized Residual | 1.003 | 0.970-1.038 | 0.842 |
| Baseline FEF_50_% | 0.968 | 0.943-0.994 | 0.017 |
| Constant |  |  | 0.164 |
| Note: ^*^AHR was transformed into three dummy variables, and the borderline AHR was administered as a reference. Model: including AHR, adherence, Unstandardized Residual, baseline FEF_50_%.  Abbreviations: AHR, Airway hyperresponsiveness; FEF_50_%, forced expiratory flow at 50% of FVC predicting. | | | |

| Supplement Table 4. Multivariable logistic regression analysis for following 2 to 3 months of poor asthma control. | | | |
| --- | --- | --- | --- |
|  | OR | 95%CI | *P* |
| Severe AHR^*^ | 9.543 | 1.468-62.023 | 0.018 |
| Adherence | 2.345 | 0.794-6.924 | 0.123 |
| Baseline FEV_1_% | 0.969 | 0.943-0.995 | 0.019 |
| Constant |  |  | 0.110 |
| Note: ^*^AHR was transformed into three dummy variables, and the borderline AHR was administered as a reference. Model: including AHR, adherence, baseline FEV_1_%.  Abbreviations: AHR, Airway hyperresponsiveness; FEV_1_%, forced expiratory volume in 1 s in predicting. | | | |
|  | | | |
|  | | | |
| Supplement Table 5. Multivariable logistic regression analysis for following 2 to 3 months of poor asthma control. | | | |
|  | OR | 95%CI | *P* |
| Severe AHR^*^ | 9.673 | 1.455-64.319 | 0.019 |
| Adherence | 2.025 | 0.683-6.006 | 0.203 |
| Baseline FEF_50_% | 0.970 | 0.953-0.988 | 0.001 |
| Constant |  |  | 0.086 |
| Note: ^*^AHR was transformed into three dummy variables, and the borderline AHR was administered as a reference. Model: including AHR, adherence, baseline FEF_50_%.  Abbreviations: AHR, Airway hyperresponsiveness; FEF_50_%, forced expiratory flow at 50% of FVC predicting. | | | |
|  | | | |
| Supplement Table 6. Multivariable logistic regression analysis for following 2 to 3 months of poor asthma control. | | | |
|  | OR | 95%CI | *P* |
| Severe AHR^*^ | 10.991 | 1.705-70.855 | 0.012 |
| Adherence | 2.257 | 0.764-6.668 | 0.141 |
| Baseline FEF_75_% | 0.981 | 0.967-0.995 | 0.010 |
| Constant |  |  | 0.514 |
| Note: ^*^AHR was transformed into three dummy variables, and the borderline AHR was administered as a reference. Model: including AHR, adherence, baseline FEF_75_%.  Abbreviations: AHR, Airway hyperresponsiveness; FEF_75_%, forced expiratory flow at 75% of FVC predicting. | | | |
|  | | | |
| Supplement Table 7. Multivariable logistic regression analysis for following 2 to 3 months of poor asthma control. | | | |
|  | OR | 95%CI | *P* |
| Severe AHR^*^ | 10.191 | 1.562-66.485 | 0.015 |
| Adherence | 2.110 | 0.713-6.244 | 0.177 |
| Baseline FEF_25-75_% | 0.976 | 0.961-0.993 | 0.004 |
| Constant |  |  | 0.226 |
| Note: ^*^AHR was transformed into three dummy variables, and the borderline AHR was administered as a reference. Model: including AHR, adherence, baseline FEF_25-75_%.  Abbreviations: AHR, Airway hyperresponsiveness; FEF25-75%, forced expiratory flow between 25-75% of FVC predicting. | | | |
