# Supplementary material for: Small airway function in predicting asthma control in preschool children
Source: Pediatr Discov. 2024 Mar 8;2(1):e46. doi: 10.1002/pdi3.46 (PMC12118259; doi:10.1002/pdi3.46)
Supplement: Supplementary file 2 — Table S8 [file PDI3-2-e46-s001.docx]

| Supplement Table 8. Linear relationships between explanatory variables and the logit transformation value of the outcome variable (poor asthma control). |
| --- |

| Variables | Wald | *P* |
| --- | --- | --- |
| Age by Ln age | 1.238 | 0.266 |
| Baseline FEV_1_% by Ln baseline FEV_1_% | 0.116 | 0.734 |
| Baseline FEF_50_% by Ln baseline FEF_50_% | 2.989 | 0.126 |
| Baseline FEF_75_% by Ln baseline FEF_75_% | 2.169 | 0.141 |
| Baseline FEF_25-75_% by Ln baseline FEF_25-75_% | 2.455 | 0.117 |
